# Fishing pressure impacts the abundance gradient of European lobsters across the borders of a newly established marine protected area

**DOI:** 10.1098/rspb.2018.2455

**Published:** 2019-01-16

**Authors:** Portia Joy Nillos Kleiven, Sigurd Heiberg Espeland, Esben Moland Olsen, Rene A. Abesamis, Even Moland, Alf Ring Kleiven

**Affiliations:** 1 Institute of Marine Research, Nye Flødevigveien 20, 4817 His, Norway; 2 Center for Coastal Research, Department of Natural Sciences, Faculty of Science and Engineering, University of Agder, 4604 Kristiansand, Norway; 3 Silliman University-Angelo King Center for Research and Environmental Management, Bantayan, Dumaguete City 6200, Philippines

**Keywords:** decapod crustacean, *Homarus gammarus*, marine protected area, catch-per-unit-effort, spillover, recreational fisheries

## Abstract

Marine protected areas (MPAs) are considered viable fisheries management tools due to their potential benefits of adult spillover and recruitment subsidy to nearby fisheries. However, before–after control–impact studies that explore the biological and fishery effects of MPAs to surrounding fisheries are scarce. We present results from a fine-scale spatial gradient study conducted before and after the implementation of a 5 km^2^ lobster MPA in southern Norway. A significant nonlinear response in lobster abundance, estimated as catch-per-unit-effort (CPUE) from experimental fishing, was detected within 2 years of protection. After 4 years, CPUE values inside the MPA had increased by a magnitude of 2.6 compared to before-protection values. CPUE showed a significant nonlinear decline from the centre of the MPA, with a depression immediately outside the border and a plateau in fished areas. Overall fishing pressure almost doubled over the course of the study. The highest increase in fishing pressure (by a magnitude of 3) was recorded within 1 km of the MPA border, providing a plausible cause for the depression in CPUE. Taken together, these results demonstrate the need to regulate fishing pressure in surrounding areas when MPAs are implemented as fishery management tools.

## 1. Introduction

Marine protected areas (MPAs), defined as sea areas where harvesting of target species is partially or fully prohibited, have been established in many regions around the world with the objectives of species conservation and management of fishery resources [1,2]. Evidence from many studies indicates that organisms targeted by fisheries increase in abundance and grow to larger body sizes inside MPAs as a direct result of protection (e.g. [3–5]).

For a protected area to be effective as a fishery management tool, the benefits of increased abundance and size inside the MPA must be exported outside. One mechanism is through spillover, or the net export of adult biomass across the borders to fished areas outside the MPA [6,7]. Spillover of target species from MPAs to adjacent fished areas is potentially mediated by both density-dependent and density-independent movements [8]. Density-dependent spillover can develop when the population density of target species increases inside the MPA, followed by an increase of competitive interactions which causes increased movement of displaced individuals to low-density areas outside the MPA [9]. Increased abundance inside the MPA coupled with increasing displacements create a decreasing gradient of abundance as one moves away from the centre of the protected area [10,11]. When these displaced individuals are captured by fishers, the spillover benefit of MPAs to fisheries is realized. Spillover can be measured by monitoring catch-per-unit-effort (CPUE) in fished areas close to the MPA borders [12]. It is well documented for multi-species, vertebrate fisheries [9,13,14], but there are fewer studies that demonstrate spillover for crustacean fisheries (see [12,15–17]).

The responses of both target species and fishers to MPA establishment are best quantified by gathering data before and after such an intervention in the same site. Studies that use this before-and-after approach for quantifying fishery effects are few and far between because of the logistic challenges such a study requires [18,19]. Also, only a few studies have been conducted on gradients of abundance across MPA borders for lobster fisheries [16,17], and to the best of our knowledge, none has been published so far with data from before and immediately after MPA establishment.

In temperate waters where MPAs are set up, crustacean response in terms of abundance can be rapid, as with the rock lobster *Jasus edwardsii* [20,21] and the spiny lobster *Palinurus elephas* [22]. A rapid increase in European lobster *H. gammarus* abundance has been observed within the Lundy no-take zone in the UK within 4 years of protection [23]. In Norway, *H. gammarus* numbers have been reported to increase by 245% inside small MPAs within 4 years of protection, compared with an increase of 87% in nearby control areas [19].

However, it is not only target species that respond to protection in MPAs. In many cases, across Europe and around the world, fishers have responded to the perceived increases in target species size and abundance inside the MPA by moving their fishing activities or increasing their fishing effort closer to the borders [24] after protected area implementation [25], a phenomenon called ‘fishing the line’ [10]. This leads to increased fishing pressure around the MPA, which can theoretically erode the benefits of protection [11].

Fishing pressure for the European lobster is high in some areas in Norway despite the continuing decline in lobster populations [26]. The fishery is dominated by recreational fishers [27]. Regulatory measures include gear number limits, minimum and maximum legal size-limit and restricted fishing periods. Notwithstanding these regulations, the lack of lobster population recovery indicates the need for better management of this fishery resource [28]. It was this impetus that drove the establishment of several MPAs with the aim of enhancing local lobster populations in the Norwegian Skagerrak coast in the early 2000s [29].

In this study, we show that the implementation of an MPA for lobsters has a simultaneous effect on both target species and the fisheries around it. We show this by (1) using before–after time series data to quantify the spatial development of an abundance gradient for lobsters (as indexed by CPUE from experimental fishing) inside and around the MPA, and (2) documenting the changes in fishing patterns that happened in the surrounding unprotected areas.

## 2. Material and methods

### (a) Subject species and study site

The European lobster (*Homarus gammarus)* is a long-lived decapod that is traditionally important to coastal communities in southern Norway. Its preferred habitats are rocky substrate or boulder fields where it can find suitable burrows to live in and defend [30]. European lobsters can grow to a total body length (TL) of up to 50 cm, and attain sexual maturity at 22–25 cm TL [31]. Individual lobsters may have limited home ranges of less than 1 km^2^ [32–34], and strong site fidelity [35]. This suggests that European lobsters do not require large MPAs to receive adequate protection from fishing [32], and their site fidelity and limited movement makes them ideal candidates for studying demographic responses to protection [36]. In southern Norway, lobsters can only be fished during a two-month season (1 October to 30 November). The total number of traps that can be deployed per fisher is limited to 10 and 100 traps for recreational and commercial fishers, respectively. In 2008, a ban on landing and trading berried (egg-bearing) females was also introduced, and the minimum legal size was increased from 24 to 25 cm TL. As of 2017, a slot limit was introduced by gazetting a maximum legal size at 32 cm TL.

**Figure 1.**
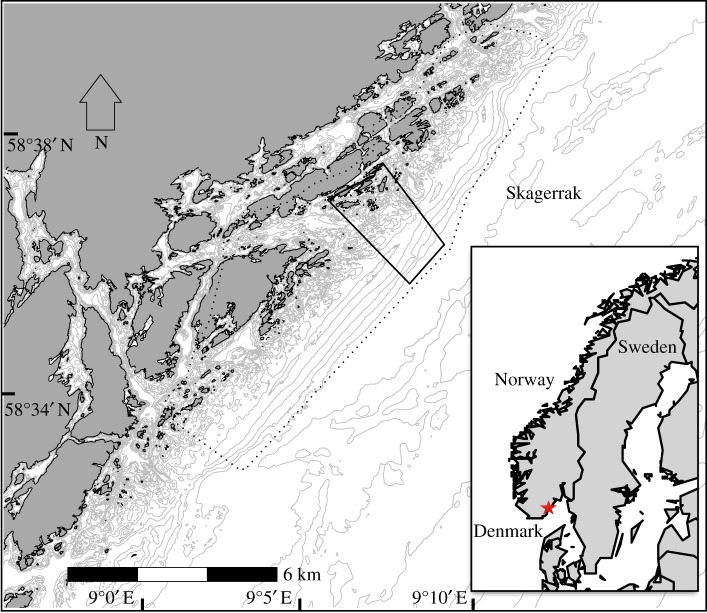
Detailed map of the Tvedestrand coast showing the MPA (box with solid line), and the study area (dashed line). Grey lines indicate depth contours. Inset: map of northern Europe indicating approximate location of study area (red star). (Online version in colour.)

The site for this study encompasses a 52.4 km^2^ area in the outer skerries of Tvedestrand municipality, with a partially protected MPA covering a water surface of about 4.9 km^2^ in the centre of the study area (figure 1). This MPA for lobsters (9°8′ 0″ E, 58°36′30″ N) was established in 2012 as the result of a process initiated by the local government, motivated by the successes in small-scale experimental MPAs in Skagerrak. Only hook-and-line-type fishing gear is allowed within the MPA. All other fishing methods that potentially catch lobsters are prohibited. The MPA is situated in an area generally classified as rocky substrate with similar topography to areas around it, with a submerged glacial moraine running parallel to the coastline. The moraine rock reef is a preferred fishing ground by the locals, and the lobster MPA site was decided after a series of consultations, hearings and discussions involving the municipal government of Tvedestrand, local organizations and scientists from the Institute of Marine Research. The outermost border of the MPA, with a depth of about 60 m, is adjacent to the rim of the Norwegian Trench which forms a natural deep-water barrier for lobster movement.

To determine the response of both lobsters and fishers to protected area establishment, two main methods were used: standardized trap surveys and fishing effort monitoring surveys.

### (b) Standardized trap surveys

Two-chambered lobster traps (90 × 45 × 40 cm) with 11.5 cm entrance diameter and closed escape vents were used for experimental fishing. The surveys were conducted yearly from 2010 to 2016 in and around the MPA to determine gradients in experimental CPUE. Similar monitoring using experimental fishing has been used in other studies to determine the effects of MPAs to adjoining fisheries [9,16,23]. Sampling was conducted at the same period every year (last week of August to first week of September), a month before the start of the lobster fishing season in Norway. Sampling effort varied throughout the course of the study (table 1). Sampling effort was doubled in 2016 to obtain better-quality data for statistical analysis. Trap locations inside and around the MPA were selected at random during the pilot survey (2010). From 2011 onwards, the sampling regime was modified slightly using topography data to maximize sampling efficiency while still maintaining randomized trap locations (random stratified). Sampling was thus limited to only those areas that (1) have a rocky bottom, (2) have a slope between 5 and 20° and a maximum depth of 35 m from the surface, and (3) are situated inside the study area (within 3 km northeast and southwest of the borders).

**Table 1.** Total number of traps deployed and total number of lobsters caught in the study area from 2010 to 2016*.*

	2010	2011	2012	2013	2014	2015	2016
number of traps deployed							
fished area	110	104	127	89	203	191	353
lobster MPA	17	39	60	55	139	88	182
total	127	143	187	144	342	279	535
number of lobsters caught							
fished area	102	39	67	55	113	94	129
females	36	14	30	31	55	52	65
males	66	25	37	24	58	42	64
lobster MPA	10	22	21	49	128	87	148
females	4	12	10	23	58	30	61
males	6	10	11	26	70	57	87
total	112	61	88	104	241	181	277

The traps were baited with frozen mackerel (*Scomber scombrus*) before deployment and attached to a marker buoy with a 40–45 m length rope. Marker buoys for each trap were individually numbered and carried information about the experimental fishing activity. Time, GPS position and depth were recorded for each trap haul. All lobsters caught were released at their capture location.

### (c) Monitoring of fishing effort

Surveys to monitor fishing effort of lobster fishers were conducted in the opening week of the fishing season, because this is when fishing effort is usually highest [26]. Fishing effort within (before implementation) and around the MPA was determined by conducting systematic boat-based total counts of all recreational and commercial lobster traps in the study area at the beginning of the lobster fishing season in 2009, 2014, 2015 and 2016. Recreational gear was easily differentiated from commercial gear, because the former has the fisher's name and address on the marker buoy, while latter has the fishing vessel registration code on the buoy. The locations of all lobster traps (marked by buoys) in the study area were recorded as GPS coordinates. These coordinates were then plotted on a map. The Euclidean distances of these points (as well as the locations of the experimental fishing traps) to the nearest border were then determined (gDistance from the R package *rgeos*). Mean distance to the borders were determined for different years. Fishing intensity was determined by generating a density map for each year with the use of kernel smoothed density analysis for point pattern data [37].

### (d) Assumptions and limitations

In using CPUE estimates obtained from experimental fishing (hereafter referred to only as CPUE) as an index for lobster abundance for this study, we assumed that lobsters have a similar catchability in baited traps that are deployed within their home range, both inside and outside the protected area. Since the habitats in and around the lobster MPA are similar, we expected that: (1) CPUE within the protected area borders will increase with increasing years of protection (rapid biological response) and (2) CPUE will be highest in the middle of the MPA due to lack of fishing mortality, creating a decreasing gradient towards the borders and outwards to the fished areas. Furthermore, data used in analyses of CPUE was also limited only to traps that were in the water for 24 h; CPUE values are thus presented as number of lobsters × trap^−1^ day^−1^.

### (e) Data analyses

To achieve parsimony in the analysis of CPUE, we formulated a zero-inflated Poisson generalized additive model [38] of the main factors (distance from border and years of protection) that influence CPUE. Depth was added as a random factor. The optimal model determined by backwards step AIC selection is as follows:

2.1 $$C_{hij}=s_{1}(B_{h},P_{i})\;+s_{2}(D_{j})+\;\beta_{k}\;+\varepsilon_{ijk}.$$

Here, the model predicted CPUE (C) for a trap at distance *h* and year *i* at depth *j* is given by the interaction between the distance from the border (*B_h_*) and years of protection (*P_i_*) as well as the depth of trap from the surface (*D_j_*). The splines (*s*_1_ and *s*_2_) are the smoothing functions modelled as a product of quadratically penalized regression spline basis functions of B, P and D with software-determined automatic smoothness estimation. *β_k_* is the model intercept and *ɛ_ijk_* is the error term (see electronic supplementary material, S1 for intercept values and GAM output). The fitted values for CPUE (at model predicted optimal depth) were obtained by using the formula:

2.2 $$\mathrm{CPUE}=\log(C_{hij}).$$

The locations of traps (longitude and latitude) were converted into metric Universal Transverse Mercator (UTM) units for use in data analyses and mapping in R. Spatial and temporal correlation was checked using auto-correlation tests (ACF), to confirm that the data were neither spatially nor temporally correlated before proceeding with the analysis. The distance from the border for each trap was calculated as the shortest distance to the nearest MPA border. To aid interpretation, the border line was designated as 0. Distance from the border was negative inside the MPA and positive outside the MPA.

Data preparation and analyses, as well as generation of figures and maps were done primarily in the R environment for statistical computing (http://www.r-project.org) using the following packages: *mgcv* [38], *pscl* [39], *splancs* [40], *rgeos* [41] and *sp* [42]. Validation for the optimal model is done by using the Pearson residual and the inspection of residual plots for the zero-inflated models (see electronic supplementary material, S1).

## 3. Results

### (a) Lobster abundance

**Figure 2.**
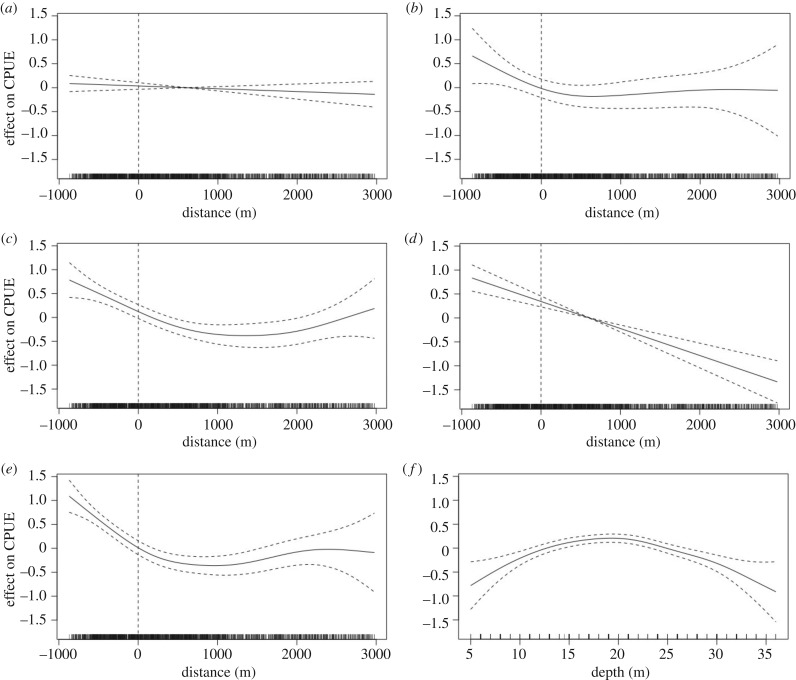
(*a*–*e*) The effect of the interaction of trap distance from MPA border and year of protection on lobster CPUE, derived from GAM. (*a*) Before MPA establishment; (*b*) 1 year after establishment; (*c*) 2 years after; (*d*) 3 years after; and (*e*) 4 years after. Vertical dashed line at 0 m represents the border. Dotted nonlinear lines are 95% confidence intervals. (*f*) Additive effect of depth on CPUE.

Depth and the interaction between distance from the border and years since protection were significantly associated with the CPUE response from the 2nd year of protection onwards (*p* < 0.0001). A decreasing nonlinear response in CPUE with distance from the MPA centre towards the borders developed after the protected area was established in 2012. The individual smoothers for distance to border per year and depth revealed the individual effect of these variables to the number of lobsters caught per trap day^−1^ (figure 2*a–f*). The smoother for depth (figure 2*f*) indicated that traps hauled from approximately 20 m depth had higher CPUE values compared to other depths, while CPUE values followed the general trend of a nonlinear decline away from MPA centre. The predictive model explained 27.4% of the variation observed in the dataset, and residual plots using predicted and residual values indicated a good model fit (see electronic supplementary material, figure S1).

**Figure 3.**
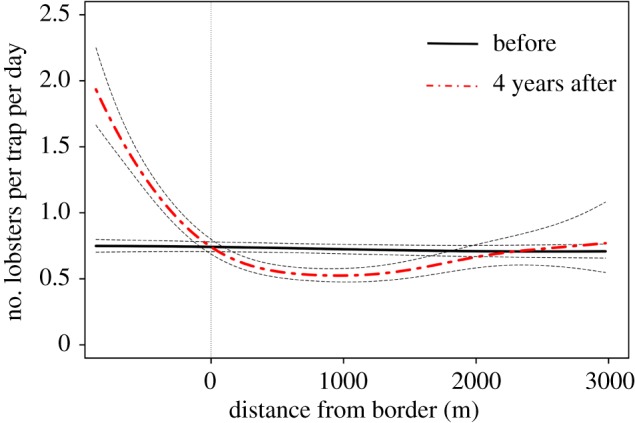
Model-predicted lobster CPUE at optimal depth (20 m below the surface) prior to (black solid line) and 4 years after protection (red dashed line). Vertical dotted line at 0 indicates MPA border. Black dashed lines are 95% confidence intervals around the prediction line.

The model prediction at the optimal depth of 20 m (figure 3) combined the results from the smoothers applied to depth, years of protection and distance to the border. The result indicated a trend of increasing CPUE inside the MPA with increased years of protection compared to the fished areas outside. The model predicted that CPUE inside the protected area had increased by a factor of 2.6 since the start of protection, from 0.748 lobsters × trap^−1^ d^−1^ (±0.049 CI) prior to protection to 1.93 lobsters × trap^−1^ d^−1^ (±0.315 CI) in 2016. However, CPUE in the adjacent fished areas between 0 and 1.5 km from the border were lower by 0.25 lobsters per trap-day compared with before-protection values. CPUE values in fished areas further away, in comparison, were similar to values before MPA establishment.

### (b) Lobster fishing intensity

**Figure 4.**
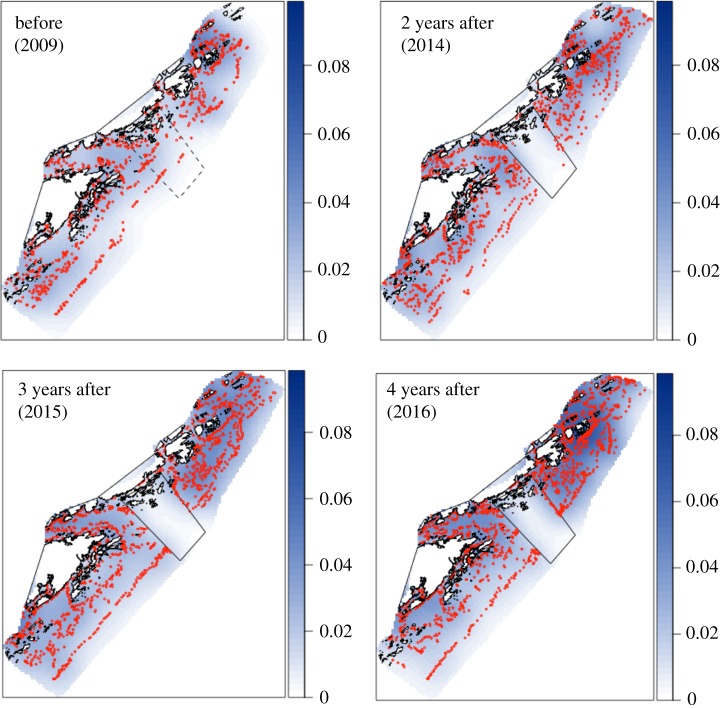
Relative intensity (blue) and location (red dots) of lobster traps in the study area during the first week of the open season from 2009 to 2016. Scale indicates number of traps observed per 1000 m^2^ (i.e. the hotspot in 2016 approx. 3 km northeast of the MPA has 8 traps per 100 000 m^2^). MPA borders are indicated as dashed lines (in 2009) and solid lines (2014–2016).

**Figure 5.**
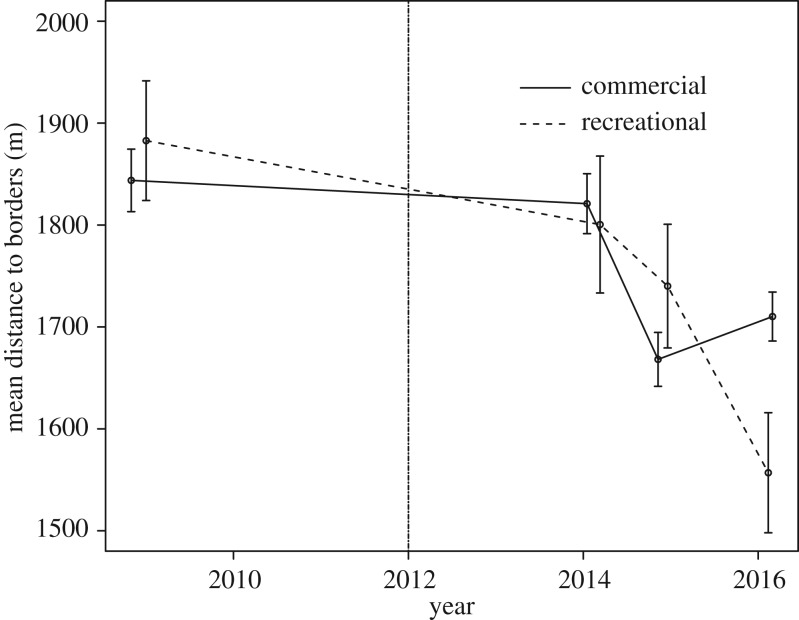
Mean distances of lobster traps by fishery type to the border from 2009 to 2016. Stippled vertical line indicates the start of protection. Error bars are ±s.e. No data are available from 2010 to 2013.

**Figure 6.**
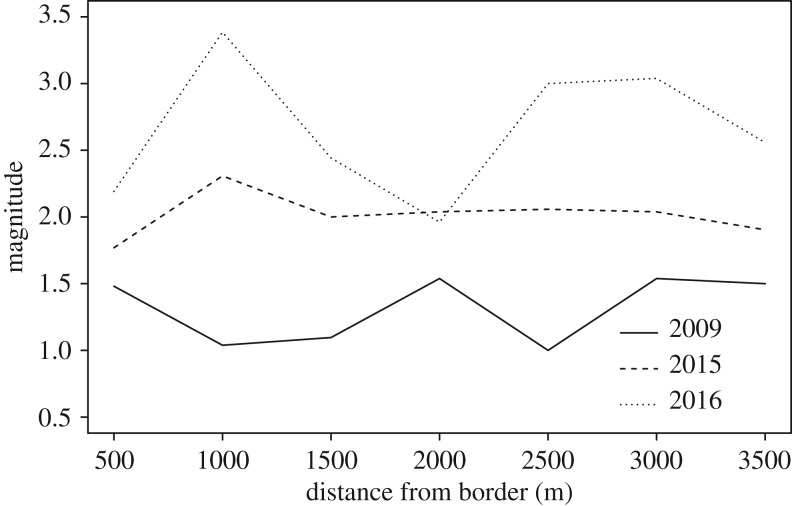
Standardized spatial distribution of fishing effort (combined for both recreational and commercial lobster fishers) before (2009) and after protection (2015 and 2016) in the fished area around the MPA. Baseline value of 1 represents 52 traps.

The number of lobster traps observed in the study area increased by 79.4% throughout the study period, from 806 traps in 2009 to 1446 traps in 2016. This increase reflects the general trend of increased recreational fishing pressure in Norway [27]. Fishing hotspots appeared and seemed to intensify over time (figure 4). Recreational traps comprised the majority of traps observed, and total number of recreational lobster traps increased with each passing year. The percentage of recreational traps increased from 72.9% of total traps observed in 2014 to 84.5% of total traps observed in 2016. More traps were being deployed closer to the MPA borders in 2016 than in 2009 (before MPA designation), and this trend was more strongly driven by recreational fishers (figure 5). In 2009, the mean distance of lobster traps from the future MPA borders for both commercial and recreational fishers was 1853.77 m ± 45.65 m SE. In 2016, the mean distance from the designated MPA border decreased to 1691.32 m ± 32.75 m SE. Closer examination of the trap data indicated that fishing pressure was not evenly distributed spatially. Trap numbers markedly increased in the fished area in 2015, with the highest increase (by more than double) noted close to the border, peaking at approximately 1 km outside the MPA (figure 6). The year after, the increase was even higher (more than triple) near the border. Trap numbers at 2500–3000 m away from the border more than doubled from 2015 to 2016.

## 4. Discussion

This empirical study showed that while lobster abundance inside an MPA can rapidly increase with protection, ‘fishing-the-line' could also quickly reduce lobster abundances close to the MPA border to levels below pre-MPA values. While lobster abundance inside an MPA had almost tripled during the first four years of MPA implementation, there was a significant decrease in lobster abundance within 1.5 km outside the MPA borders. A simultaneous threefold increase in fishing pressure within the same distance range and location provides a likely explanation for this decrease. Our findings may have broad consequences for designing future MPAs, and indicate that adaptive management of fishing effort in areas surrounding MPAs are required to ensure that their conservation and fishery goals are reached.

Protection had an immediate effect on the abundance of lobsters within the borders in our study MPA: a gradient was visible after only one year. This rapid response of lobsters to protection has been noted in earlier studies in other areas [19,20,22]. However, to the best of our knowledge, our study is the first to document that a significant nonlinear gradient of *H. gammarus* abundance can develop as early as 2 years after MPA implementation. Gradients of abundance that develop due to protection are expected to eventually extend outside the protected area borders, and eventually benefit the neighbouring fished areas through the mechanism of spillover [43]. The steepness of this gradient depends on many factors, among them the mobility of the target species, the size of the MPA, how fast the population recovers and fishing pressure outside the borders [11,44].

Our results corroborate findings from simulations made by Pérez-Ruzafa *et al.* [11] which indicated that even small MPAs can positively influence the abundance of target species both inside and outside the MPA borders irrespective of the fishing pressure outside the protected area; the higher the fishing mortality, the more limited is the spatial extent of the increased abundance outside the borders. The same study indicated that declining gradients towards the baseline values were always present except when there was no fishing at all. It did not predict a depression at the border, however, unlike the earlier simulation made by Kellner *et al*. [10], which suggested that a dip in fish density and CPUE can occur at the reserve border under conditions of concentrated fishing pressure or ‘fishing the line'. Furthermore, this dip is expected to be more pronounced for species with limited mobility compared with species that are highly mobile [10].

The CPUE gradient we observed in our study conforms to the model prediction of Kellner *et al.* (high fishing pressure on species with limited mobility) [10], and is very similar to the field observations of Goñi *et al*. [16] on fisheries CPUE for spiny lobster *Palinurus elephas* and Kay *et al.* [17] on research CPUE for spiny lobster *Panulirus interruptus*. Furthermore, our observation of values that are lower than the before-protection data right outside the border indicate the strong impact of increased fishing pressure on lobster abundances in the fished area. Although we did not conduct a correlation analysis, a probable link between increased fishing pressure near the border and depressed CPUE values the year after was indicated by the data.

In a commercial lobster fishery, Goñi *et al.* [16] attributed the depression in the CPUE gradient they observed adjacent to protected areas to depletion associated with the concentration of fishing effort at the borders. Howarth *et al.* [45] also suggested increased fishing activity near the marine reserve borders and high levels of fishing mortality to be the cause of CPUE decline immediately within and outside a marine reserve. In the present study, fisheries CPUE (both commercial and recreational) would have been useful as a measure to quantify fishing pressure, but collection of such data requires logistics that we did not have. Furthermore, the steep increase in number of lobster traps observed in the study area over time reflects the increasing popularity of recreational fishing in southern Norway. A more than threefold increase in number of lobster traps near the border indicate that this is becoming a preferred fishing area for most recreational fishers.

While lobster abundance is increasing inside the protected area, the intensified fishing clearly had a strong effect on the fished side of the border, effectively reducing the abundance of lobsters immediately around the protected area. This concentrated fishing effort near the border can be interpreted as an affirmation that the MPA is perceived to function positively from fishers' point of view, because recreational fishers that ‘fish the line' are not motivated by an increase in revenue, but rather, are driven by a mixture of catch expectation and the value of the fishing experience itself [46].

Earlier modelling studies have suggested that, over time, intense fishing the line may act to diminish the effect of protection on lobster abundance within an MPA, because it can encourage emigration towards newly available habitats right outside the border [10,11]. The effect will be more pronounced over time, especially if the fishing pressure intensifies [11]. This implies that strict monitoring and regulation of fishing effort around MPAs should be co-implemented with MPA establishment.

In summary, we show that increased fishing pressure around a newly established MPA impacted on the development of the expected spillover benefit represented by a gradient of abundance across MPA borders. Precautionary management of fishing effort, especially in the early years of implementation, may be necessary to secure the long-term conservation and fishery effects of small lobster MPAs. A marine protected area is a good tool for local fisheries management [47], but it is a spatial management tool that requires time in order to work. Moreover, it should not be used in isolation but should rather be implemented together with other targeted measures to curb overfishing. Managers thus need to consider displacement of effort and the expected shift and increase in intensity of fishing activities around the borders in the early phase whenever an MPA is implemented as a local fisheries management tool. In the MPA planning process, inclusiveness and transparency are important when weighing the potential future benefits of protection (e.g. spillover effect) against the overall ability of the management system to curb overfishing in the adjacent fishery.

## Supplementary Material

GAM residual plots and summary outputs

## Supplementary Material

GAM residual plots and summary outputs

## Supplementary Material

GAM residual plots and summary outputs

## Supplementary Material

GAM residual plots and summary outputs

## Data accessibility

Data available from the Dryad Digital Repository: https://dx.doi.org/10.5061/dryad.rs39059 [48].
