# Supplementary figures and images for: Fishing pressure impacts the abundance gradient of European lobsters across the borders of a newly established marine protected area
Source: Proc Biol Sci. 2019 Jan 16;286(1894):20182455. doi: 10.1098/rspb.2018.2455 (PMC6367164; doi:10.1098/rspb.2018.2455)

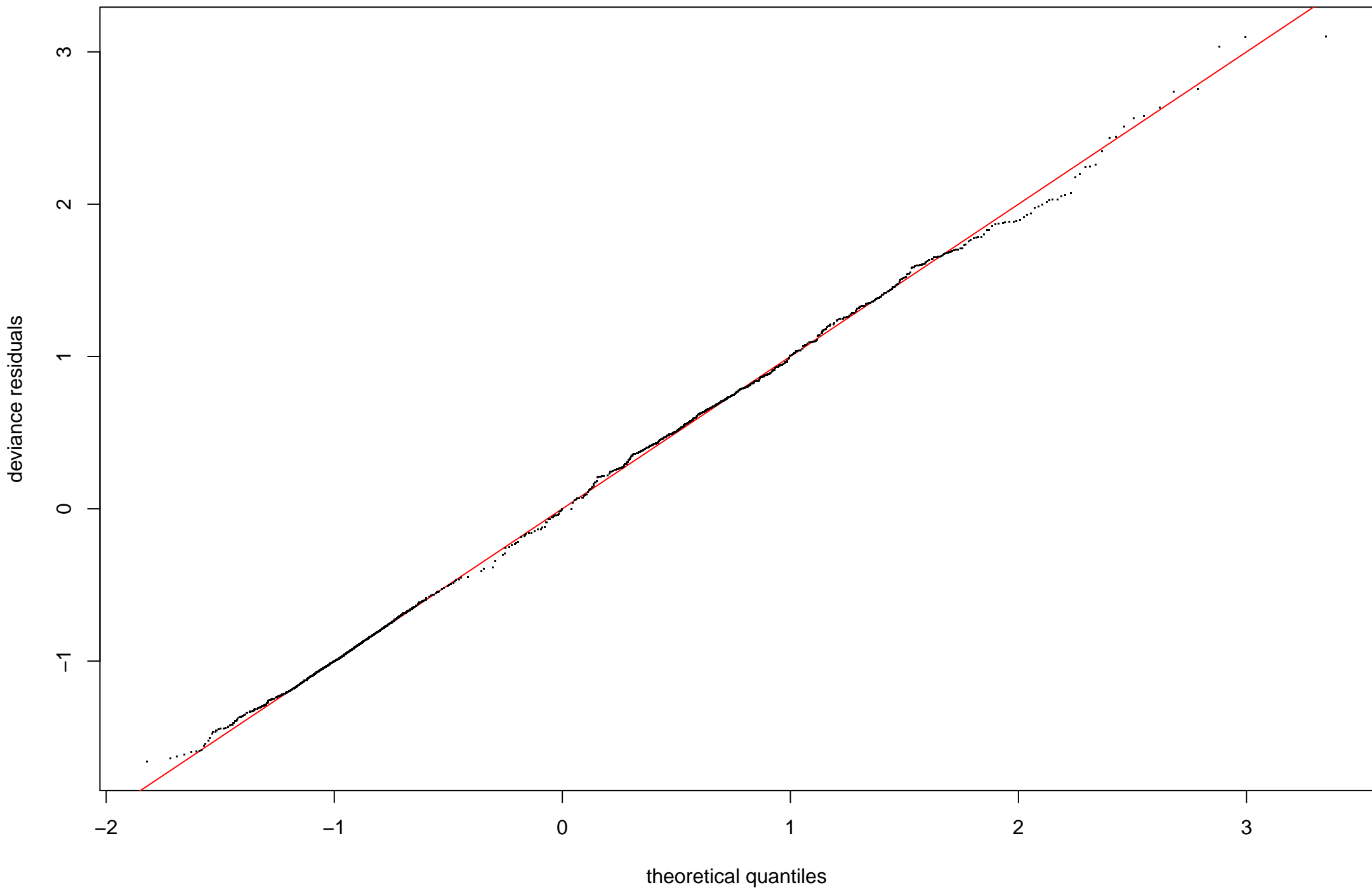

Supplement: GAM residual plots and summary outputs [file rspb20182455supp1.pdf]

Resids vs. linear pred.

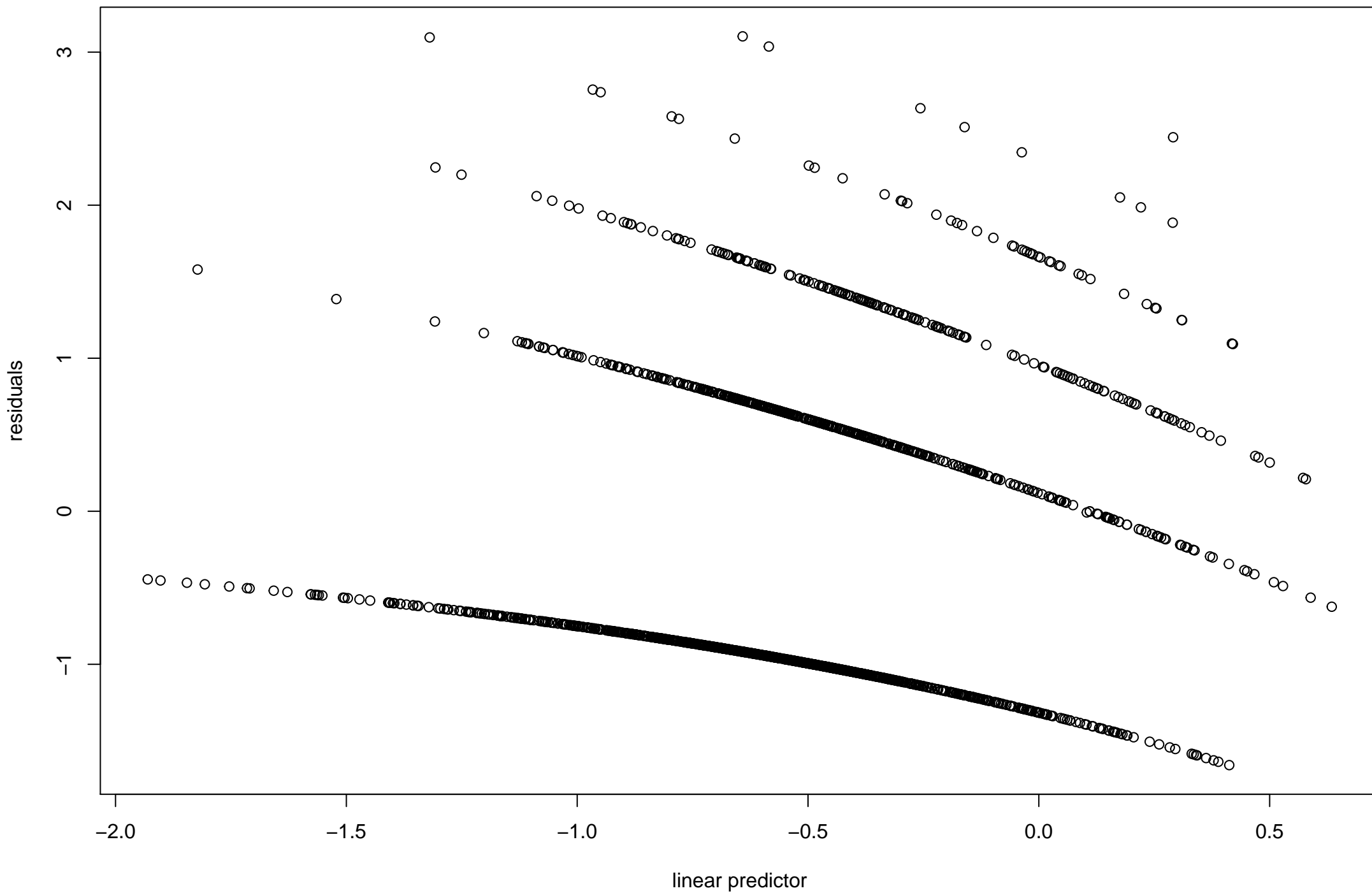

Supplement: GAM residual plots and summary outputs [file rspb20182455supp2.pdf]

**Histogram of residuals**

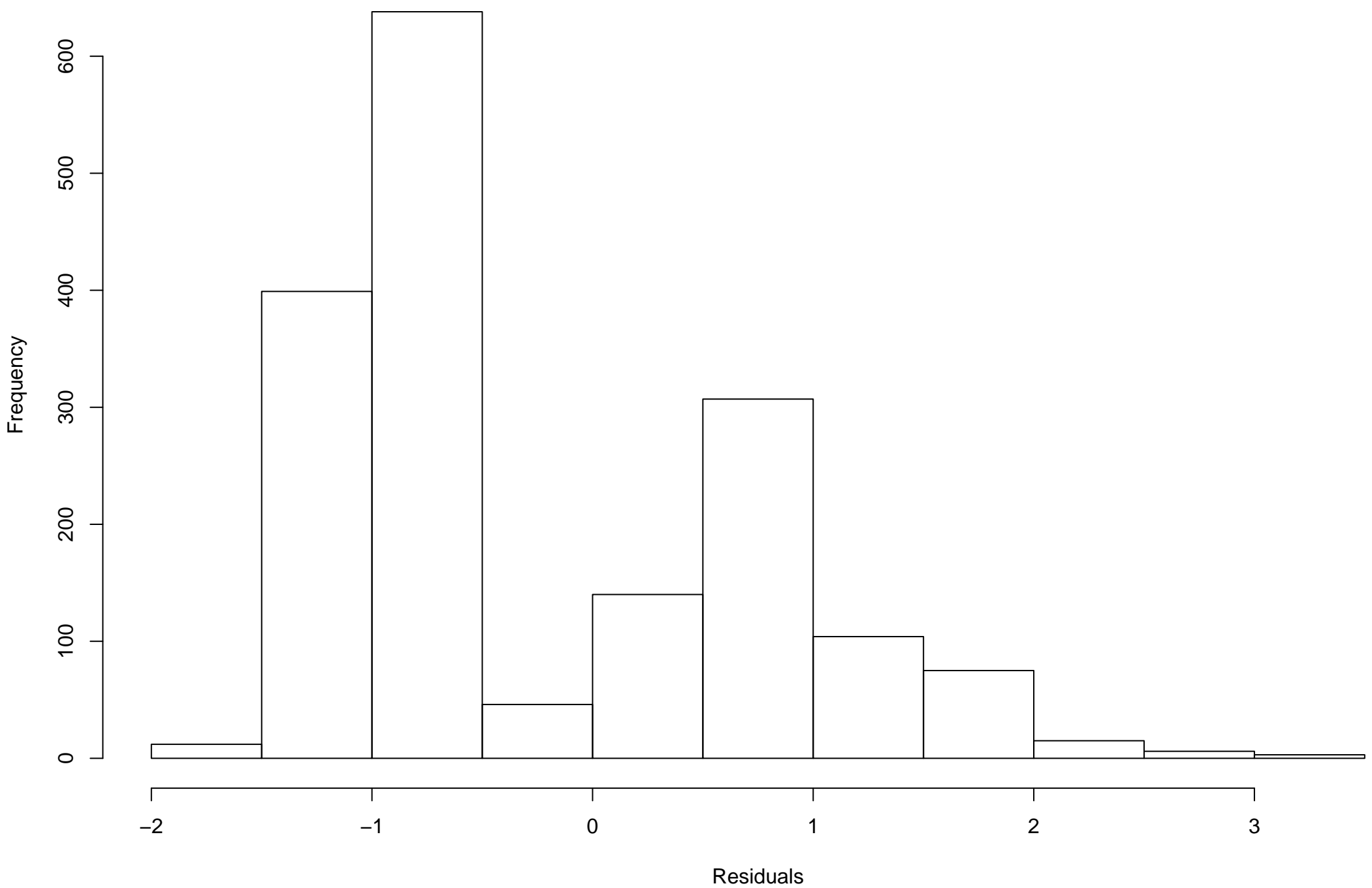

Supplement: GAM residual plots and summary outputs [file rspb20182455supp3.pdf]

Response vs. Fitted Values

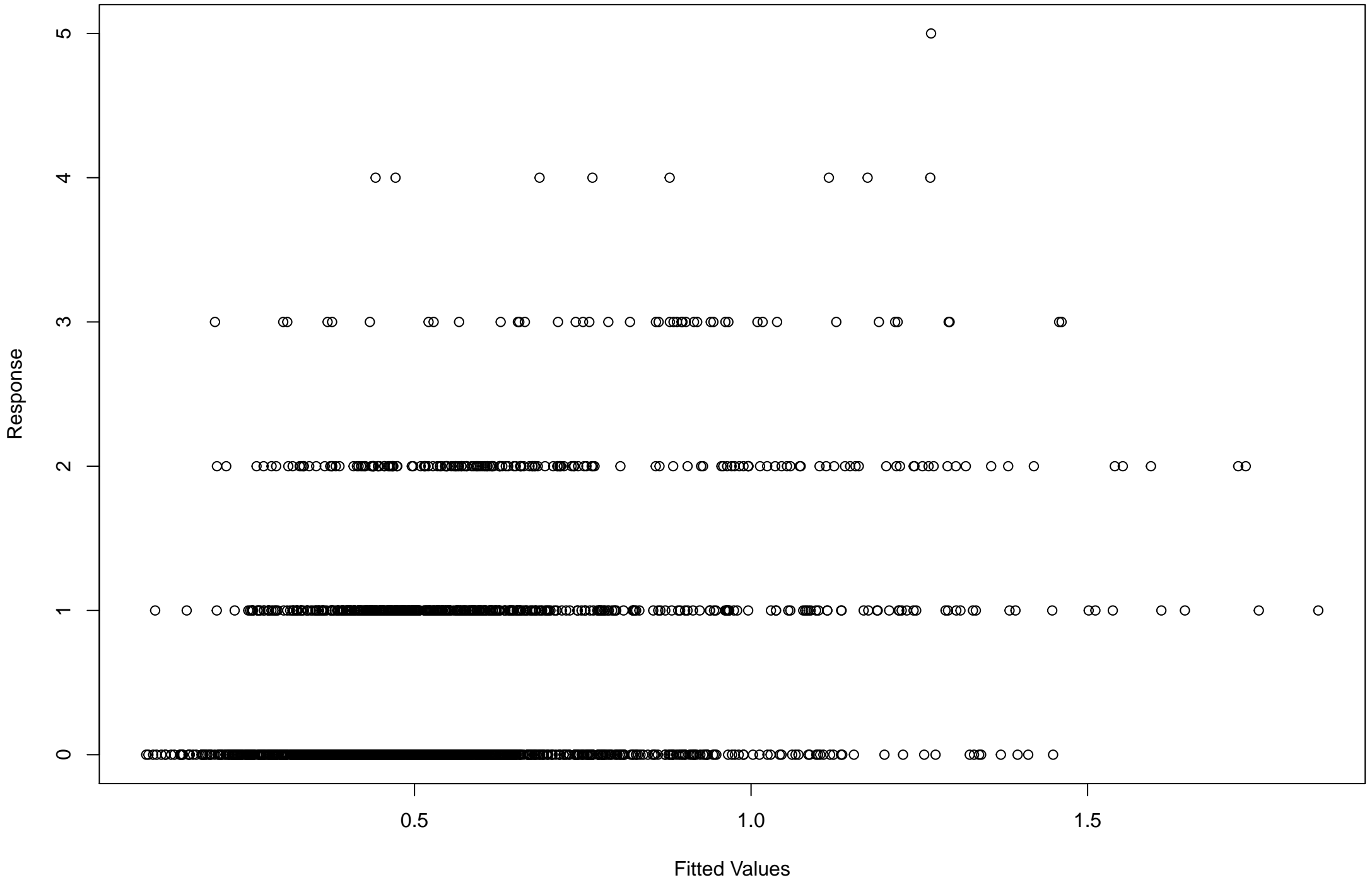

Supplement: GAM residual plots and summary outputs [file rspb20182455supp4.pdf]
